# Age-appropriate vaccination coverage and its associated factors for pentavalent 1-3 and measles vaccine doses, in northeast Ethiopia: A community-based cross-sectional study

**DOI:** 10.1371/journal.pone.0218470

**Published:** 2019-08-16

**Authors:** Tefera Alemu Marefiaw, Muluken Azage Yenesew, Kebadnew Mulatu Mihirete

**Affiliations:** 1 Amhara Public Health Institute, Public Health Emergency Management Directorate, Dessie, Ethiopia; 2 Department of Environmental Health, School of Public Health, College of Medicine and Health Sciences, Bahir Dar University, Bahir Dar, Ethiopia; 3 Department of Epidemiology and Biostatistics, School of Public Health, College of Medicine and Health Sciences, Bahir Dar University, Bahir Dar, Ethiopia; University of Campania, ITALY

## Abstract

**Background:**

In Ethiopia, there are limited studies on age-appropriate vaccinations that children received at the recommended specific ages. Therefore, we assessed age-appropriate vaccinations coverage and its associated factors among children 12 to 23 months of age in Menz Lalo district, northeast Ethiopia.

**Methods:**

A community-based cross-sectional study was conducted in Menz Lalo district from March to April/2018 among 417 mothers/caregivers with children 12 to 23 months of age using simple random sampling technique. Data were collected using a pretested structured questionnaire. Information about children’s vaccination status was collected from vaccination cards. Age-appropriate vaccination coverage was measured using World Health Organization vaccination schedule recommendation. Data was entered into Epi-Info^7^ software and exported to SPSS-20 for analysis. Four consecutive logistic regression models were performed to identify factors associated with age-inappropriate vaccinations. A P-value of ≤ 0.05 was considered to state statistically significant associations.

**Results:**

Age-appropriate vaccination coverage was **39.1%** (95% CI: 34.3 to 44) for *pentavalent* 1, **36.3%** (95% CI: 31.6 to 41.5) for *pentavalent* 2, **30.3%** (95% CI: 25.6 to 35) for *pentavalent* 3 and **26.4%** (95% CI: 21.7 to 31) for measles vaccine doses. Age-inappropriate *pentavalent* 1–3 vaccinations was associated with being male sex (AOR: 0.47, 95% CI: 0.29–0.74), lack of telephone (AOR: 2.2, 95% CI: 1.4–3.6), lack of usual caretaker (AOR: 2.6, 95% CI: 1.3–5.2), unplanned pregnancy (AOR: 1.9, 95% CI: 1.1–3.5), missing pregnant women’s conference (AOR: 2.7, 95% CI: 1.3–5.7), decreasing birth order (AOR: 0.34, 95% CI: 0.17–0.68) and insufficient knowledge (AOR: 2.7, 95% CI: 1.6–4.4).

**Conclusion:**

The proportions of age-appropriate vaccination coverage were low in the study area. Modifiable factors were associated with age-inappropriate vaccinations. Vaccination interventions should consider identified modifiable factors to improve age-appropriate vaccinations coverage.

## Introduction

Globally, significant progress in up to date vaccination coverage has been made since 1980, resulting in the prevention of an estimated 2 to 3 million deaths every year from vaccine-preventable diseases (VPDs). Despite this overwhelming success, an estimated 1.5 million children are continued to die every year from VPDs; mainly in developing countries [1]. This requires to maximize high vaccination coverage with timely administration of vaccines to attain the full benefits of vaccinations; since untimely high vaccination coverage will lead to false assumptions of disease protection [2,3]. Thus, a child should receive all vaccinations within the recommended age and intervals to achieve maximal protection from VPDs [4,5]. Moreover, according to the World Health Organization (WHO) and researchers’ recommendation, each vaccine doses should be given at defined ages, according to the schedule, and delay is undesired [6–9].

In Ethiopia, vaccination is one of the strategies under the government health policy to improve child health by the administration of a vaccine to help the immune system develop protection from disease [10]. Consequently, *pentavalent* and measles vaccines are mandatory vaccines that have been set goals to increase their coverage above 95% in the Health Sector Transformation Plan, which is part of the Second Growth and Transformation Plan of Ethiopia [11]. Accordingly, *pentavalent* 1–3 and measles vaccine doses are scheduled to be administered at 6 weeks, 10 weeks, 14 weeks and 9 months of age respectively [6,10].

Giving a vaccine dose at less than the minimum recommended age to start vaccine and the minimum four-week interval may lessen the antibody response due to sub-optimal seroconversion rate and it should be repeated if the vaccine is administered greater than four days before the minimum age. On the other hand, lengthening the interval between doses of vaccines leads to higher antibody levels, but increased vulnerability to VPDs [4,12–14]. Besides, delayed vaccination will end up with an altered sequence of vaccinations, which in turn causes non-specific effects of vaccines with negative consequences on childhood morbidity and mortality [15,16]. Moreover, delayed vaccination is found to be a risk factor for pertussis, measles and invasive Haemophilus Influenzae type b diseases [17–19]. Thus, the recommended vaccination schedule is designed to protect infants and children early in life, when they are most vulnerable and before they are exposed to VPDs and reduces the risk of having VPDs from unvaccinated children as a result of herd immunity [13,14,20].

Globally, studies showed that few children received all recommended vaccine doses on time, even with high up-to-date coverage [8,9,21]. Similarly, in Sub-Sahara African countries, researchers observed a substantial delay in timely vaccination receipt [7,22,23]. However, in Ethiopia, studies so far have focused solely on full vaccination coverage [24–27] and studies that examined vaccination timeliness are very scarce. There is also limited information on factors associated with age-inappropriate vaccinations in the country. *Pentavalent* and measles vaccine doses are reliable parameters for measuring age-appropriateness of childhood vaccinations. Therefore, we assessed age-appropriate vaccination coverage and its associated factors for *pentavalent* 1–3 and measles vaccine doses in Menz Lalo district of Amhara region, northeast Ethiopia.

## Methods

### Study design and period

A community-based cross-sectional study was conducted to assess age-appropriate vaccinations coverage and its associated factors from March to April/2018.

### Study setting and population

The study was conducted in Menz Lalo district, North Shoa zone, Amhara region, which is located at a distance of 274 kilometers to the northeast of the capital city of Ethiopia, Addis Ababa. The district has one urban and seven rural *kebeles* (the lowest administrative units with an estimated size of 1000 households). Based on the 2007 national census, the estimated population of the district in 2017/18 was 40,781. Of which, 20,594 (50.3%) were females and 1,101 (2.7%) were children between 12 to 23 months of age [28]. In the district, there are three health centers and eight health posts that provide primary health care services to the community including vaccination for children. This makes on average one health center for an estimated 13,594 populations and one health post for an estimated 5,098 populations in the study area. In the study area, the vaccination program is coordinated by one EPI officer at the district level who leads implementation of the vaccination program in all health centers and satellite health posts. Each health center has a designated vaccination room, which provides vaccination service to children and managed by a full-time and well trained EPI focal person. Similar to the health centers, each satellite health post which is staffed by at least two health extension workers provides vaccination for children and undertakes a house to house children identification and registration, vaccination defaulter tracing and awareness creation in the community. Moreover, vaccination service is being delivered as an outreach service at least once per month to reach those children residing beyond 5 km from the static health facilities. The measles vaccine has been opened provided that a sufficient number of children exist to prevent open dose vial wastage. Each household in the *kebele* has *family folder* with a unique household identification number in the health posts. The health extension workers employed in that particular health post (*kebele*) conduct a house to house visit at least once per month to update the information’s about the household while having the *family folder* at hand. So, even though a child was born at home or wherever he/she is vaccinated; be it in static or outreach sites; finally, he/she will be registered into his/her parent’s *family folder* by HEWs. Partners are also actively collaborating with the government in EPI program implementation in the area.

All children aged 12 to 23 months with their mothers/caregivers living in Menz Lalo district were the source population. Mothers/caretakers who have a vaccination card with written records of vaccination dates were included in the study and critically ill mothers/caretakers who were unable to respond were excluded from the study.

### Sample size and sampling technique

The sample size was determined using a single population proportion formula by considering the following assumptions: 5% margin of error, 95% confidence level, and a 50% age-appropriate vaccination coverage, since there was no previous timeliness study in the country. After adding a 10% non-response rate, we obtained a final sample size of 423 children with their mothers/caregivers. All eligible children in the entire district (eight *kebeles*) were considered in the study. Firstly, the list of all eligible children (962) aged 12–23 months was taken from all health posts *family folder* and vaccination registers. All the *family folders* were updated within the past two years at least once per month. Moreover, to be sure that no eligible child is missed from the sampling frame, we have cross-checked the list of eligible children obtained from the health posts *family folder* with the vaccination registers in the health posts and health centers. In such a way, we prepared a complete list of all eligible children in the district containing information about the name of a child, his parent’s full name, households Unique Identification Number and *sub-kebele/got*. Then proportional to size allocation was made to determine the required sample size from each *kebele*. Finally, simple random sampling technique was employed to select the required number of children from each *kebele* using the listed children as a sampling frame.

### Data collection

Data was collected using structured pretested Amharic version questionnaire through face to face interview. The questionnaire had questions of socio-demographic and economic factors, maternal health service utilization, vaccination status of a child and reasons given by mothers/caregivers for none, early or late vaccination. Age-appropriate vaccination schedule questions were developed from WHO recommended schedule [6]. Information about children’s vaccination status was collected from children’s vaccination card. The questionnaire was first prepared in English and then translated to the Amharic language.

Eight health professionals participated as data collectors and supervisors. Data collectors addressed each child’s house based on the household’s unique identification number, parent name and *sub-kebele/got* which was given by the principal investigator. If the mother/caregiver was not found at the first visit, a second visit was conducted and all were addressed.

### Data quality assurance

Before the actual data collection, a pre-test was done on 5% of the study participants out of the study area and modification was made on vague terms and questions. Training was given for data collectors and supervisors to enable them to have a common understanding of the objectives of the study and each of the questions in the questionnaire. Data were checked for completeness, consistency, accuracy and clarity daily. Daily supervision was done by the supervisors and the principal investigator.

The validation of the questionnaire was done to assess the reliability of questions using Cronbach’s alpha (α). A total of 11 items were used to measure knowledge about vaccination and the internal consistency estimate of the reliability of test items was found in the good range 0.8 ≤ α < 0.9 (α = 0.853).

### Data analysis

Data completeness was checked on a daily bases and entered into Epi info^7^ software and exported to SPSS 20 for data analysis. Once the data was cleaned and coded, descriptive statistics were computed to describe the data. The outcome variable (age appropriateness of each vaccination) was dichotomized as age-appropriate and age-inappropriate. Bivariate and multivariate logistic regression analyses were used to identify factors associated with age-inappropriate vaccination of children. The chi-square goodness of fit test was checked for each variable before the regression analysis. The variance inflation factor (VIF) was also calculated to check multicollinearity between independent variables before the multivariate regression analysis. The VIF was found in the range between 1.06 and 3.9, which is below the recommendation for acceptable levels of VIF in published article (<5) [29]. A variable with a P-value of ≤ 0.2 in the bivariate analysis was considered to be a candidate variable for multivariate logistic regression analysis model to control confounding effect. Backward stepwise regression method was also used to control confounding effect between independent variables. A P-value of ≤ 0.05 was considered to state statistically significant association between independent variables and age-inappropriate vaccinations. Crude and adjusted odds ratios with 95% confidence interval were computed to observe the strength of association between the outcome variable and independent variables.

### Operational definitions

**A *pentavalent* vaccine or 5-in-1 vaccine**: is a combination vaccine with five individual vaccines conjugated into one, intended to actively protect people against five major infections: diphtheria, tetanus, pertussis (whooping cough), hepatitis B and Haemophilus influenzae type b (Hib).

**Age-appropriate vaccination (timely)**: was measured if a child was vaccinated ***within one month*** after the minimum age to administer the dose as recommended by WHO.

**Age-inappropriate vaccination (untimely)**: was measured if a child was vaccinated earlier and/or delayed than the recommended age (Table 1).

**Table 1:** Operational definition in relation to WHO & national vaccination schedule.

*Vaccine*	*WHO recommendation* (6)		*Operational definition*	
	*Minimum age*	*Minimum interval*	*Delayed*	*Early*
Pentavalent 1	6 weeks		>10 week	<42 day
		4 weeks		
Pentavalent 2	10 weeks		>14 week	< 70 day
		4 weeks		
Pentavalent 3	14 weeks		>18 week	< 98 day
Measles	9 months	4 weeks	> 10 months	< 270 days

**Delayed vaccination**: was measured as not having received the recommended vaccine doses within one month beyond the minimum age (Table 1). Accordingly, the *pentavalent* 1–3 and measles vaccine doses were categorized as delayed at >70, >98, and >126 and >300 days respectively.

**Early vaccination**: doses given before the minimum age (Table 1).

**Up to date vaccination**: the proportion of 12–23 month old children who are vaccinated with *pentavalent* 1–3 and measles vaccine doses regardless of the time of vaccination.

**Invalid dose**: doses given more than 4 days before the minimum acceptable age or the minimum acceptable interval between doses (ACIP recommendation [12]).

**Valid dose**: doses given up to 4 days before the minimum age or the minimum interval between doses

**Sufficient knowledge**: Eleven knowledge questions were asked and score greater than the mean was classified as sufficient.

**Favorable attitude**: When respondents positively reacted to at least three out of the four attitude questions regarding vaccinations, we classified as having favourable attitude.

**Family folder**: is a file folder at health post which has adopted family as a unit for the provision of family-based health services (preventive, promotive & environmental health) and also a file for each member of the family.

**Usual caretaker**: If the index child was living with his mother/caregiver throughout his one year of life, we classified the child as having a usual caretaker. In the meantime, if the index child was living without his mother/caregiver at least for one month, while he was in the first year of age, we classified as not having a usual caretaker.

**Pregnant women’s conference**: is a monthly pregnant women’s meeting held at each kebele/health post regarding their pregnancy, childbirth, postnatal care and other health related issues.

**Index child**: 12 to 23 months old child that was randomly included in the study.

**Caregiver**: is the most responsible person that provides care for the index child whose biological mother couldn’t provide intimate care.

**Kebele**: is the smallest administrative unit of Ethiopia, which contains an average of 1,000 households.

**Health post**: is the smallest unit of health facility at the lowest level of administration (kebele) providing basic health services to an average of 1,000 households.

## Results

### Socio-demographic characteristics of respondents and their children

A total of 417 mothers/caregivers who had children aged 12 to 23 months were interviewed with a response rate of 98.6%. Of all respondents, 392(94%) were mothers and 25(6%) were caregivers. Three hundred seventy-six respondents (90%) were living in a rural area and 403 (96.6%) were self-employed. The median (±SD) age of mothers/caregivers was 29(±8.1) years, which ranges from 18 to 52 years. One hundred sixty-one respondents (39.1%) were not able to read and write. The mean monthly household income of respondents was 1800 Ethiopian birr (±1022 SD) and 58% of the respondents had an income in the range of 1000 to 2500 birr per month. The proportion of male children was 227(54.4%) and 60% of children were born at health facilities (Table 2).

**Table 2:** Socio-demographic characteristics of respondents and their children in Menz Lalo district, northeast Ethiopia, 2018 (N = 417).

*Variable*	*Category*	*Frequency*	*Percent*
Residency	Rural	376	90.2
	Urban	41	9.8
Respondent	Mother	392	94
	Caregiver	25	6
Sex of child	Male	227	54.4
	Female	190	45.6
Place of birth	Health facilities	249	59.7
	Home	168	40.3
Maternal occupation	Self-employed	403	96.6
	Government employed	14	3.4
Usual caretaker	Yes	333	79.9
	No	84	20.1
Paternal education	Cannot read & write	110	26.4
	Read & write	194	46.5
	Primary (1–8)	72	17.3
	Secondary (9–12) & above	41	9.8
Maternal/caregivers education	Cannot read and write	163	39.1
	Read and write	143	34.3
	Primary(1–8)	69	16.5
	Secondary (9–12) & above	42	10.1
Pregnancy status	Unplanned	115	27.6
	Planned	302	72.4
Family size	< 5 member	149	35.7
	≥ 5 member	268	64.3
Distance to vaccination site	< 30 minute	253	60.7
	> 30 minute	164	39.3
Marital status	Married	383	91.8
	Others	34	8.2
Season of birth	Summer	127	30.5
	Autumn	93	22.3
	Winter	96	23
	Spring	101	24.2
Maternal/caregivers age	15–20 year	45	10.8
	20–30 year	191	45.8
	30–40 year	119	28.5
	40–52 year	62	14.8
Birth order	1^st^ birth order	79	18.9
	2^nd^ birth order	67	16.1
	3^rd^ birth order	62	14.9
	4^th^ birth order	88	21.1
	≥ 5^th^ & above	121	29
Monthly income in Ethiopian birr	<1000 ETB	70	16.8
	1000–2500 ETB	243	58.3
	>2500 ETB	104	24.9
Pregnant women’s conference participation	Not participated	255	61.2
	≤ 2 Participation	112	26.9
	≥ 3 Participation	50	12
ANC follow up	No follow up	100	24
	≤ 2 follow up	139	33.3
	≥ 3 follow ups	178	42.7
TT status	No dose received	152	36.5
	1 dose received	98	23.5
	≥ 2 doses received	167	40
PNC service utilization	Not received	360	86.3
	At least once	57	13.7
Knowledge about vaccination	Insufficient	193	46.3
	Sufficient	224	53.7
Attitude towards vaccination	Unfavorable	13	3.1
	Favorable	404	96.9
*Pentavalent* 1 age appropriateness	Age appropriate	162	38.8
	Age inappropriate	255	61.2
Two parent household	One parent	69	16.5
	Two parent	348	83.5
Mode of transportation	Foot	394	94.5
	Horse	23	5.5
Paternal occupation	Government employed	19	4.6
	Self employed	398	95.4
Telephone availability	Not available	207	49.6
	Available	210	50.4

Moreover, 27.6% of children were born from unplanned pregnancy and 30.5% were delivered in the summer season. Besides, 20.1% of children were living without their mothers/caregivers at least for one month during their one year of life. Sixty-one percent of mothers didn’t attende pregnant women’s conference at all; however, 42.7% of them had received at least three antenatal care follow-ups (Table 2).

### Age-appropriate vaccinations

Up to date vaccination coverage for *pentavalent* 1–3 and measles vaccine doses were 99.3% (95% CI: 98.3 to 100), 97.1% (95% CI: 95.4 to 98.6), 92.6% (95% CI: 89.9 to 95.2) and 82.7% (95% CI: 78.9 to 86.6) respectively. However, only **39.1%** (95% CI: 34.3 to 44), **36.3%** (95% CI: 31.6 to 41.5), **30.3%** (95% CI: 25.6 to 35) and **26.4%** (95% CI: 21.7 to 31) were vaccinated age-appropriately for *pentavalent* 1–3 and measles vaccine doses respectively. The proportion of antigens received earlier than the recommended national schedule was 6.8% (95% CI: 4.6 to 9.2), 5.9% (95% CI: 3.7 to 8.1), 5.2% (95% CI: 3.1 to 7.7) and **51.3%** (95% CI: 46.4 to 56.5) for *pentavalent* 1–3 and measles vaccine doses respectively. The magnitudes of delayed *pentavalent* 1–3 and measles vaccination were 54.1% (95% CI: 49.3 to 58.9), 57.8% (95% CI: 52.8 to 62.2), 64.5% (95% CI: 59.8 to 69.4) and 22.3% (95% CI: 18 to 26.9) respectively. Invalid dose coverage for *pentavalent* 1–3 was 5.1%, 4.7%, and 4.4% respectively (Table 3).

**Table 3:** Timeliness of vaccination among children aged 12–23 months in Menz Lalo district, northeast Ethiopia, 2018.

Timeliness	Pentavalent 1	Pentavalent 2	Pentavalent 3	Measles
**Early**	28(6.8)	24(5.9)	20(5.2)	177(51.3)
**Age Appropriate**	162(39.1)	147(36.3)	117(30.3)	91(26.4)
**Delayed**	224(54.1)	234(57.8)	249(64.5)	77(22.3)
**Total**	414(100)	405(100)	386(100)	345(100)

**Fig 1:**
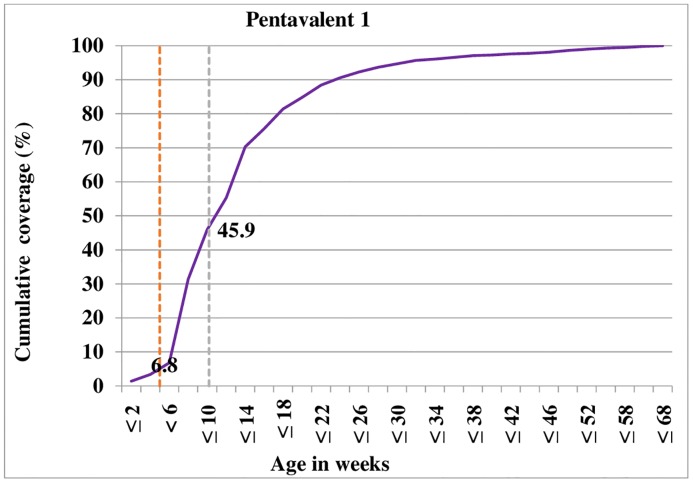
Timing of pentavalent -1 vaccination against children’s age, 2018.

**Fig 2:**
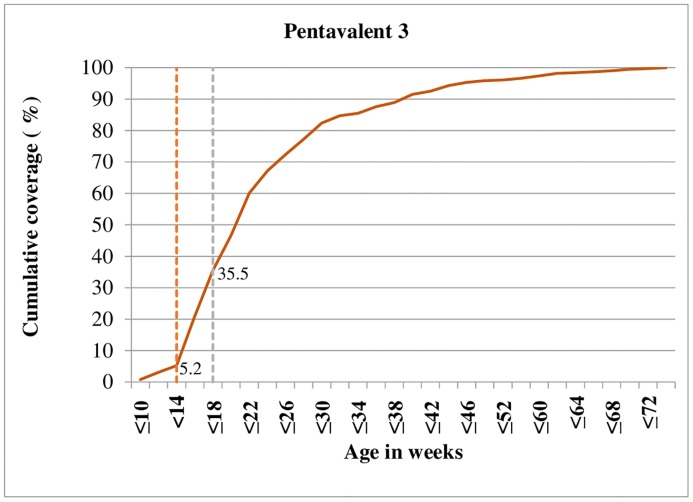
Timing of pentavalent-3 vaccination against children’s age, 2018.

**Fig 3:**
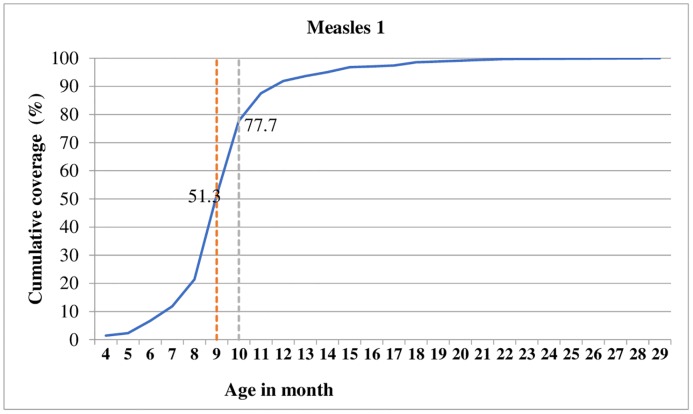
Timing of measles vaccination against children’s age, 2018.

Figs 1–3 shows cumulative vaccination coverage for each vaccine doses at a specified age. We plotted two dash lines on each figure to show the proportion of children vaccinated early (before the first vertical line), age-appropriately (in between the two vertical lines) and delayed (after the second vertical line).

Consequently, 6.8% of children have vaccinated the first dose of *pentavalent* vaccine before the recommended six weeks of age. Only 39.1% of children received the first dose of *pentavalent* vaccine at the appropriate age of 6 to 10 weeks. Apart from these, about 54.1% of children have vaccinated the first dose of *pentavalent* vaccine after 10 weeks of age (Fig 1). Similar age-inappropriate trend was seen for *pentavalent* 3 vaccination, with only 30.3% received their *pentavalent* 3 dose in between 14 to 18 weeks as recommended by WHO childhood vaccination schedule. About 5.2% of children were given *pentavalent* 3 dose before 14 weeks. Moreover, the majority (64.5%) of children received their *pentavalent* 3 dose later than 18 weeks; although the vaccine was scheduled to be administered at 14 weeks of age (Fig 2). Moreover as depicted in Fig 3, the majority (51.3%) of children have vaccinated the first dose of measles vaccine before nine months of age.

**Fig 4:**
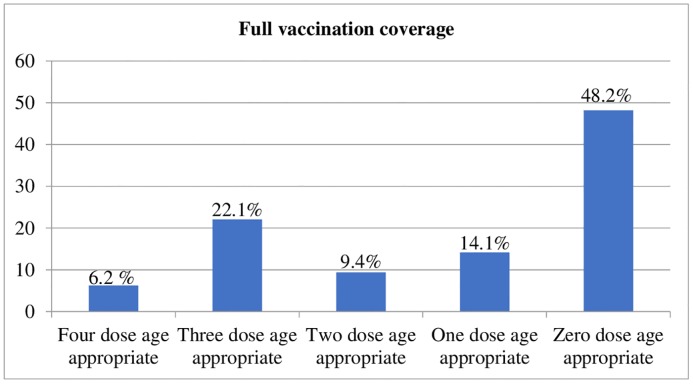
Proportion of age-appropriate vaccine doses among the four studied vaccine doses, 2018.

Overall, only 6.2% of children received all the four studied vaccine doses at their appropriate age. Nearly half (48.2%) of children didn’t receive any of the four vaccine doses on time (Fig 4). The median delay was 34 days for measles and 35.5, 41 and 44 days for *pentavalent* 1–3 doses respectively. Median early delivery time for doses received earlier than the recommended schedule was 14.5, 16, 16.5 and 23 days for *pentavalent* 1–3 and measles vaccine doses respectively.

### Reasons for none or age-inappropriate measles vaccination

Mothers or caregivers were asked about the reasons for none or age-inappropriate vaccination of their children. The reason given by mothers/caregivers for early reception of measles vaccine was health workers’ appointment before nine months of age to prevent open dose vial wastage (54.3%). Nearly 13% of delay and none vaccination was due to insufficient number of children to open the measles vial. Other reasons include mothers/caregivers being too busy on appointment day (8.6%), forgotten vaccination appointment 23 (7%), inconvenient time or day of vaccination (6.1%) and others (4.6%) (Table 4).

**Table 4:** Frequency of reasons for none or age-inappropriate measles vaccination among children 12–23 months of age in Menz Lalo district, northeast Ethiopia, 2018 (N = 326).

Reasons	Frequency	Percent
Health workers appointed me before 9 month	177	54.3
Postponed due to insufficient children to open the vial	42	12.9
Mothers/caregivers being too busy	28	8.6
Forgotten or no vaccination appointment	23	7.0
Day or time, place of vaccination inconvenient	20	6.1
Mothers/caregivers or child get ill on the appointment day	11	3.4
Vaccine stock outs	10	3.1
Other reasons/multiple responses	15	4.6
Total	326	100

Regarding specific reasons for age-inappropriate *pentavalent* 1 vaccination, the most frequent reasons of delay were religious exemption (52.5%), fear of side effects after injection (22.7%) and mothers/caregivers being too busy (12.2%) (Table 5).

**Table 5:** Reasons for none or age-inappropriate pentavalent 1 dose vaccination among children 12–23 months of age, in Menz Lalo district, northeast Ethiopia, 2018(N = 255).

Reasons	Frequency	Percent
Religious exemption	134	52.5
Fear of side effects	58	22.7
Health professionals vaccinated the child early	31	12.2
Mothers/caregivers being too busy	31	12.2
Forgotten or no vaccination appointments	13	5.1
Others/multiple responses	6	2.4

### Factors associated with age-inappropriate vaccinations

In *pentavalent* 1 bivariate analysis model; maternal/caregivers educational status, paternal educational status, place of birth, child’s birth order, telephone availability, usual caretaker, sex of child, pregnancy status, season of birth, pregnant women’s conference participation, knowledge about vaccination, ANC follow up and PNC service utilization was found to be the predictors of age-inappropriate *pentavalent* 1 dose vaccination at P-value of ≤0.2. However in multivariate analysis; birth order, telephone availability, usual caretaker, sex of the child, pregnancy status, pregnant women’s conference participation and knowledge about vaccination remains statistically significant predictors of age-inappropriate *pentavalent* 1 dose vaccination (Table 6).

**Table 6:** Factors associated with age-inappropriate pentavalent 1 vaccination among children 12–23 months of age in Menz Lalo district, northeast Ethiopia, 2018 (N = 417).

Variables	Category	Pentavalent 1 age appropriateness		COR (95% CI)	AOR(95% CI)
		Age-inappropriate	Age-appropriate		
Sex of child	Female	121	106	1	1
	Male	134	56	2.096(1.39–3.14)	2.13(1.35–3.45) *
Telephone availability	Not available	157	50	3.6 (2.36–5.45)	2.2(1.35–3.6)*
	Available	98	112	1	1
Pregnancy status	Unplanned	90	25	2.98 (1.81–4.91)	1.938(1.07–3.5)**
	Planned	165	137	1	1
Usual caretaker	No	71	13	4.42 (2.35–8.3)	2.55(1.25–5.16)*
	Yes	184	149	1	1
Pregnant women conference participation	Not participated	182	73	4.84 (2.5_9.2)	2.74(1.34–5.66)*
	≤ 2 participation	56	56	1.94 (0.97–3.88)	1.83(0.85–4)**
	≥3 participation	17	33	1	1
Birth order	1^st^ birth order	40	39	0.401(0.22–0.72)	0.336(0.167–0.676)*
	2^nd^ birth order	35	32	0.43(0.22–0.79)	0.52(0.251–1.07)***
	3^rd^ birth order	37	25	0.578(0.304–1.1)	0.648(0.312–1.34)***
	4^th^ birth order	56	32	0.684(0.38–1.23)	0.757(0.386–1.48)***
	≥5^th^ & above	87	34	1	1
Knowledge about vaccination	Insufficient	151	42	4.1(2.7–6.4)	2.68(1.62–4.42)*
	Sufficient	104	120	1	1

*P-value < 0.01,

** P-value ≤ 0.05,

*** P-value > 0.05

The odds of having age-inappropriate *pentavalent* 1 vaccination among female children were two times (AOR: 2.13, 95% CI: 1.35–3.45) higher when compared to male children. Similarly, the odds of having age-inappropriate *pentavalent* 1 vaccination among children who didn’t have usual caretaker were 2.5 times higher compared to their counter parts. However, the odds of age-inappropriate *pentavalent* 1 vaccination were decreased substantially with decreasing birth order. Likewise, the availability of telephone in the household was found to decrease the odds of age-inappropriate *pentavalent* 1 vaccination with more than two folds (AOR: 2.2, 95% CI: 1.35–3.6). On the other hand, failure to participate in pregnant women’s conference increases the odds of age-inappropriate *pentavalent* 1 vaccination by 2.7 times compared to mothers who have 3 or more participation in pregnant women’s conference (AOR: 2.74, 95% CI: 1.34–5.66). Also, unplanned pregnancy was found to increase the odds of age-inappropriate *pentavalent* 1 vaccination with two folds (AOR: 1.938, 95% CI: 1.07–3.5) (Table 6).

In the second bivariate analysis model; residency, maternal/caregivers educational status, paternal educational status, place of birth, child’s birth order, telephone availability, usual caretaker, sex of child, pregnancy status, pregnant women’s conference participation, knowledge about vaccination, family size, TT status, distance to vaccination site and ANC follow up were found to be the predictors of age-inappropriate *pentavalent* 2 vaccination at P-value of ≤ 0.2. But in multivariate analysis, only maternal/caregiver’s educational status, telephone availability, usual caretaker, sex of child, pregnant women’s conference participation, knowledge about vaccination, and PNC service utilization independently associated with age-inappropriate *pentavalent* 2 dose vaccination (Table 7). Accordingly, 82.4% of children from mothers/caregivers with insufficient knowledge about vaccination were received their *pentavalent* 2 vaccination age-inappropriately compared to 49.6% of their counterparts (AOR = 3.17, 95% CI: 1.85–5.45). Three fourth (74.5%) of mothers who didn’t participate on pregnant women’s conferences were found to vaccinate their children age-inappropriately as compared with 38% of mothers who have three or more participation in pregnant women’s conference (AOR: 3.82, 95% CI: 1.73–8.42). Like to the first model, the variables male sex, absence of usual caretaker and lack of telephone in the household were found to be the contributing factors for age-inappropriate *pentavalent* 2 vaccination. Decreasing birth order is also found to be a protective factor from age-inappropriate *pentavalent* 2 vaccination (AOR: 0.26, 95% CI: 0.13–0.54).

**Table 7:** Factors associated with age-inappropriate pentavalent 2 vaccination among children 12–23 months of age in Menz Lalo district, northeast Ethiopia, 2018 (N = 417).

Variables	Category	Pentavalent 2 age appropriateness		COR (95% CI)	AOR (95% CI)
		Age inappropriate	Age appropriate		
Sex of child	Male	129 (56.8)	98 (43.2)	0.45(0.3–0.69)	0.44(0.27–0.7)*
	Female	141 (74.2)	49(25.8)	1	1
Usual caretaker	No	74(88.1)	10(11.9)	5.17(2.58–10.36)	3.22(1.49–6.96)*
	Yes	196(58.8)	137(41.1)	1	1
Pregnant women’s conference participation	Not participated	190(74.5)	65(25.5)	4.76(2.52–9.01)	3.06(1.49–6.3)*
	≤2 participation	61(54.5)	51(45.5)	1.95(-0.98–3.85)	1.94(0.9–4.17)***
	≥3 participation	19(38)	31(62)	1	1
Birth order	1^st^ birth order	41(51.9)	38(48.1)	0.33(0.18–0.59)	0.26(0.13–0.54)*
	2^nd^ birth order	39(58.2)	28(41.8)	0.42(0.22–0.79)	0.45(0.21–0.95)**
	3^rd^ birth order	40(64.5)	22(35.5)	0.55(0.28–1.1)	0.55(0.26–1.2)***
	4^th^ birth order	57(64.8)	31(35.2)	0.55(0.3–1.02)	0.55(0.27–1.1)***
	≥5^th^ & above	93(76.9)	28(23.1)	1	1
Knowledge about vaccination	Insufficient	159(82.4)	34(17.6)	4.76(3–7.5)	3.3(1.95–5.58)*
	Sufficient	111(49.6)	113(50.4)	1	1
Telephone availability	Not available	163(78.7)	44(21.3)	3.56(2.32–5.47)	2.12(1.28–3.5)*
	Available	107(51)	103(49)	1	1

*P-value < 0.01,

** P-value ≤ 0.05,

*** P-value > 0.05

In pentavalent 3 bivariate analysis model; telephone availability, usual caretaker, sex of the child, pregnancy status, knowledge about vaccination, ANC follow up, pregnant women’s conference participation, TT status and season of birth were the associated factors with age-inappropriate *pentavalent* 3 vaccination at P-value of ≤ 0.02. However in multivariate analysis; telephone availability, usual caretaker, sex of the child, knowledge about vaccination and ANC follow up remain statistically significant predictors of age-inappropriate *pentavalent* 3 vaccination (Table 8). Consequently, male children were 52.4% less likely to receive their *pentavalent* 3 vaccination age-inappropriately as compared to their counterparts (AOR: 0.476, 95% CI: 0.289–0.786). Ninety-three percent of children who didn’t have usual caretakers were vaccinated age-inappropriately as compared to 66.7% of children who have usual caretaker (AOR: 4.26, 95% CI: 1.7–10.7). Similarly, 88.1% of mothers/caregivers with insufficient knowledge about vaccination were found to vaccinate their children age-inappropriately as compared to 58% of mothers/caregivers with sufficient knowledge (AOR: 3.38, 95% CI: 1.9–6.0). The absence of telephone in the household is also found to be a contributing factor for age-inappropriate *pentavalent* 3 vaccination (AOR: 1.97, 95% CI: 1.18–3.3). The other identified factor in *pentavalent* 3 model is maternal ANC follow up. In this case, children from mothers having three or more ANC follow up had a better chance of being vaccinated age-appropriately than children from low or no ANC follow up.

**Table 8:** Factors associated with age-inappropriate pentavalent 3 vaccination among children 12–23 months of age in Menz Lalo district, northeast Ethiopia, 2018 (N = 417).

Variables	Category	Pentavalent 3 age appropriateness		COR (95% CI)	AOR (95% CI)
		Age inappropriate	Age appropriate		
Sex of child	Male	150(66)	77 (34)	0.51(0.33–0.81)	0.476(0.289–0.786)*
	Female	150(79)	40(21)	1	1
Usual caretaker	No	78(92.9)	6(7.1)	6.5(2.74–15.37)	4.26(1.7–10.7)*
	Yes	222(66.7)	111(33.3)	1	1
ANC follow up	No follow up	77(77)	23(23)	2.27(1.3–3.95)	1.07(0.56–2.04)***
	≤ 2 follow up	117(84.2)	22(15.8)	3.6(-2.09–6.23)	2.28(1.24–4.17)*
	≥ 3 follow ups	106(59.6)	72(40.4)	1	1
Knowledge about vaccination	Insufficient	170(88.1)	23(11.9)	5.34(3.2–8.9)	3.38(1.9–6.0)*
	Sufficient	130(58)	94(42)	1	1
Telephone availability	Not available	172(83.1)	35(16.9)	3.14(1.99–4.97)	1.97(1.18–3.3)*
	Available	128(61)	82(39)	1	1

*P-value < 0.01,

** P-value ≤ 0.05,

*** P-value > 0.05

In measles model, we run bivariate logistic regression on all the predictors and only ANC follow up and *pentavalent* 1 dose age appropriateness was found to be the predictors of age-inappropriate measles vaccination (P-value ≤ 0.2); however, none of them remains significant in multivariate analysis model (Table 9).

**Table 9:** Bivariate analysis of factors associated with age-inappropriate measles vaccination among children 12–23 months of age in Menz Lalo district, northeast Ethiopia, 2018 (N = 417).

Variables	Category	Measles age appropriateness		COR (95% CI)
		Age-inappropriate	Age-appropriate	
ANC follow up	No follow up	87	13	2.33(1.2–4.56)
	≤ 2 follow up	107	32	1.16(0.694–1.95)
	≥ 3 follow-ups	132	46	1
Pentavalent 1 age appropriateness	Age appropriate	118	44	0.6(0.379–0.969)
	Age inappropriate	208	47	1

## Discussion

Both high coverage and timely administration of vaccination are useful to attain the full benefits of vaccination. We obtained the up to date vaccination coverage above the 90% target set by WHO, except measles which was 82.7%. This figure is in line with different study findings that were conducted in various parts of the country [25,27] and with a recent study from sixteen European countries [30], where the primary diphtheria-tetanus-pertussis (DTP) containing vaccine coverage ranges from 89.1% to 98.2%. However in the present study; only 39.1%, 36.3%, 30.3%, and 26.4% were vaccinated at their appropriate age for *pentavalent* 1–3 and measles vaccine doses respectively. This could be because the national vaccination policy, which had targeted to achieve a more than 95% *pentavalent* and measles vaccination coverage in 2020, had simply focused on the up to date coverage, irrespective of the time of vaccination [10,11]. This finding is also in line with previous reports from developing and developed countries [7,15,21,23,31,32] that reported a high proportion of age-inappropriate vaccinations despite high up to date vaccination coverage. This calls for age appropriateness of vaccination to be considered as another indicator of vaccination program performance to ensure timely administration of vaccination for children. In contrast to this, a study done in Zhejiang province of China found a comparably high proportion of age-appropriate measles vaccination [33]. Nonetheless, as the national experience of Italy tells us [34,35], measles elimination, of course to all VPDs elimination, will remain unrealized dream unless the vaccination program is being monitored against its schedule with strong political commitment.

In the present study age-appropriate vaccination coverage was not only low, but also it declines as one goes from *pentavalent* 1 to *pentavalent* 3 and measles vaccine doses. This might be due to increased maternal/caregiver’s workload with other domestic activities while the child gets older and thereby can’t remember vaccination appointments of a child. The other possible explanation could be the occurrence of side effects like fever, pain or swelling following prior dose vaccination that will not motivate mothers/caregivers for the next appointment.

Despite several studies worldwide didn’t consider the possibility of a too early administration of vaccines, this study found 6.8%, 5.9% and 5.2% early administration of antigens for *pentavalent* 1–3 doses. This result is lower than the finding from Burkina Faso [15]. However, we didn’t found any mother/caregiver who brought her child for vaccination earlier than her appointment day. This indicates as vaccinators were appointing mothers/caregivers and administering vaccines before the schedule for a variety of reasons. This might be the reason for the difference from Burkina Faso finding. Besides, in contrary to other studies [7,22]; we found a high proportion (51.3%) of children vaccinated before nine months of age for measles. For this, mothers/caregivers were asked the reason behind and nearly all of them blamed vaccinators for contributing to it by appointing them before nine months of age. In this regard, the measles multi-dose vial has been opened once or twice per month, provided that sufficient numbers of children were found in the vaccination site; be it in a static or outreach setting.

Besides, we found invalid dose coverage of 5.1%, 4.7% and 4.4% for *pentavalent* 1–3 doses respectively. According to the Advisory Committee on Immunization Practice (ACIP) recommendation [12], these invalid doses leads to low seroconversion rates thereby less child protection from vaccine-preventable disease and it should be repeated.

Overall, only 6.2% of children had received all the four studied vaccine doses at their appropriate age. This is low when compared with studies from Uganda (18%), Gambia (36.7%), Kenya (38%) and India (31%) [22,23,31,36]. As already discussed above, less or no attention for vaccination timeliness and the absence of timeliness indicator in the Ethiopian vaccination program might be the possible reason. In addition to this, 48.2% of children hadn’t received any of the four vaccine doses on time. These results in pooling of unprotected or inadequately protected susceptible individuals at the study area which will contributes for the occurrence of outbreaks of vaccine-preventable diseases.

Each vaccine-specific model produced a unique combination of risk factors with only sex, telephone availability, usual caretaker and knowledge about vaccination as a risk factors common to all *pentavalent* models. This demonstrates that difference exists in factors associated with timeliness of each vaccine doses and the combined fully age-appropriate vaccination. This distinction is very important to understand the characteristics of children who are more likely to experience age-inappropriate vaccinations and to intervene accordingly.

The risk of age-inappropriate *pentavalent* 1–3 vaccination was increased by more than two folds in children with insufficient maternal/caregivers knowledge about vaccination. Even though it was with the up to date model, similar correlation was reported from southeast and northern Ethiopia [27,37]. Similarly, the odds of age-inappropriate *pentavalent* 1–3 vaccination was increased by more than two, three and four folds respectively in children who did not have usual caretakers. This is best explained by the fact that mothers/caregivers might not handover their child’s vaccination appointment day for the immediate caretaker in situations where the mother/caregiver is not the child’s caregiver. Being male child was also found to be a protective factor from age-inappropriate *pentavalent* 1–3 vaccination by 53%, 56%, and 52.4% respectively. This result is in line with previous studies done in northwest Ethiopia [38,39]. But in a study done in southeast Ethiopia, Kenya and China, no correlation was detected between sex and delayed *pentavalent*1, *pentavalent* 3, or measles vaccine [27,31,40]. This is largely attributed to the belief that prohibits taking a female infant out of the door before 80 days of birth in our study area. However, it is also possible that our finding represents a sex preference towards a male. Now a day it becomes usual to remind mothers/caregivers through their telephone about their vaccination date to improve timely vaccination uptakes. In all *pentavalent* models, our finding also indicates that the tendency of age-inappropriate *pentavalent* 1–3 vaccination among households without telephone was more than two folds as compared to their counterparts. This implies that reminding mothers/caregivers with a phone has significant role to increase timely vaccination uptakes, especially in the case of outreach settings and multi-dose vial vaccines to prevent open dose vial wastage. However, the variable telephone availability was not significantly associated with vaccination timeliness in a study done in Malawi and the United States of America [41,42].

As compared to their counterparts, the odds of children from unplanned pregnancy were two times more likely to take their *pentavalent* 1 vaccination age-inappropriately (AOR: 1.94, 95% CI: 1.07–3.5). This finding is in argument with previous up to date reports in Ethiopia [38]. Also, failure to participate in pregnant women’s conference increases the odds of age-inappropriate *pentavalent* 1 & 2 vaccinations with more than 2.7 folds when compared to mothers who had three or more conference participation (AOR: 2.74, 95% CI: 1.34–5.66). This might be because mothers during the conference would receive health education about vaccination and related issues. Even though it is not surprising to have a delay of subsequent dose after delay in initial dose, our study revealed that age-inappropriate *pentavalent* 1 vaccination is a strong predictor of age-inappropriate subsequent dose vaccinations. However, we didn’t found a significant association between *pentavalent* 1 dose age appropriateness and measles dose timeliness. Moreover, the absence of a predictor variable in the measles advanced model could be explained by the high rate of early (51.3%) and late (12.2%) administration of the measles vaccine by vaccinators. This is to prevent open dose vial wastage due to multi-dose vial presentation of the measles vaccine. Thus, to what extent the mother/caregiver wishes timely vaccination, her child didn’t get vaccinated unless otherwise on that specific measles vaccination day. This automatically leads to age-inappropriate measles vaccination. That is why all of our predictor variables turned out to be none significant in the measles model.

Finally, our study is not without limitation. Firstly, our study participants might have introduced recall bias in remembering the frequency of maternal health service utilization factors and some of the reasons for none or age-inappropriate vaccination especially if the child is aged near to two years. Additionally, over-reporting opinions and behaviours that are congruent with values deemed socially acceptable and under-reporting those deemed socially undesirable might have been introduced in some of our study questions. Nonetheless, we provided detailed information that will aid clinicians and public health officials in low vaccination timeliness settings in directing resources towards a goal of protecting children from vaccine-preventable disease.

## Conclusion

The proportions of age-appropriate vaccination coverage were low in the study area. The predictors of age-inappropriate *pentavalent* 1–3 vaccinations were lack of telephone in the household, absence of usual caretaker, being female child, unplanned pregnancy, not participating in pregnant women’s conference, increased birth order and insufficient maternal/caregivers knowledge about vaccination. The chief reason for age-inappropriate measles vaccination was its multi-dose vial presentation. Our finding suggests for the Ministry of Health to integrate vaccination timeliness (early, age-appropriate and delayed) into Demographic Health Information System (DHIS_2_) database to measure its performance. Any vaccination intervention should consider identified modifiable factors to improve age-appropriate vaccination. Furthermore, health facility vaccinators should focus on timeliness of vaccination to minimize early and delayed administration of vaccines.

## Supporting information

S1 Dataset Raw data used in the analysis for this published article. (SAV)

## Abbreviations

ACIP: Advisory Committee on Immunization Practice
ANC: Antenatal Care Follow up
DHIS_2_: Demographic Health Information System
EPI: Expanded Program on Immunization
HEWs: Health Extension Workers
PNC: Postnatal Care
SD: Standard Deviation
TT: Tetanus Toxiod Vaccination
VPDs: Vaccine Preventable Diseases
WHO: World Health Organization
